# Targeted, noninvasive blockade of cortical neuronal activity

**DOI:** 10.1038/srep16253

**Published:** 2015-11-06

**Authors:** Nathan McDannold, Yongzhi Zhang, Chanikarn Power, Costas D. Arvanitis, Natalia Vykhodtseva, Margaret Livingstone

**Affiliations:** 1 Department of Radiology, Brigham and Women’s Hospital, Harvard Medical School, Boston, Massachusetts, USA; 2 Department of Neurobiology, Harvard Medical School, Boston, Massachusetts, USA

## Abstract

Here we describe a novel method to noninvasively modulate targeted brain areas through the temporary disruption of the blood-brain barrier (BBB) via focused ultrasound, enabling focal delivery of a neuroactive substance. Ultrasound was used to locally disrupt the BBB in rat somatosensory cortex, and intravenous administration of GABA then produced a dose-dependent suppression of somatosensory-evoked potentials in response to electrical stimulation of the sciatic nerve. No suppression was observed 1–5 days afterwards or in control animals where the BBB was not disrupted. This method has several advantages over existing techniques: it is noninvasive; it is repeatable via additional GABA injections; multiple brain regions can be affected simultaneously; suppression magnitude can be titrated by GABA dose; and the method can be used with freely behaving subjects. We anticipate that the application of neuroactive substances in this way will be a useful tool for noninvasively mapping brain function, and potentially for surgical planning or novel therapies.

Methods to record and map brain function have advanced dramatically in the last decade, and the ability to map activity noninvasively with functional MRI has revolutionized the study of the human brain. Techniques to modulate brain function have also gained importance in neuroscience. Direct electrical stimulation and localized injection of neuroactive substances have long been a mainstay of neurobiology research, and optogenetic techniques now permit the modulation of neuronal activity at a cellular level. Direct current stimulation is now an important clinical tool for disrupting brain circuits but the invasiveness of inserting wires into the brain is a major drawback to this approach. Transcranial magnetic stimulation does permit noninvasive neuronal modulation, but the spatial resolution of this approach is very coarse. We have developed an entirely noninvasive technique that permits local, targeted, neuronal modulation that could potentially be used in humans.

Ultrasound can be focused into the brain to induce heating or localized mechanical effects without affecting overlying structures; this method has been investigated as a noninvasive therapeutic tool for more than 70 years^1^. Focused ultrasound (FUS) can transiently disrupt the blood-brain barrier (BBB) at precisely targeted regions^2^. The effect is achieved by combining ultrasound bursts with a microbubble ultrasound contrast agent. The microbubbles concentrate the mechanical effects of the acoustic wave on the vasculature, resulting in opening of the tight junctions and initiation of transcellular transport^3^. FUS-induced BBB disruption is being evaluated for targeted drug delivery for brain tumors, neurodegenerative diseases, and other CNS disorders^4^.

The ability to noninvasively disrupt the BBB at targeted locations can also enable the delivery of neurotransmitters or other neuroactive agents. In this study we investigated the feasibility of temporarily inhibiting brain activity in a targeted region via FUS-induced BBB disruption followed by intravenous administration of γ-Aminobutyric acid (GABA). GABA is an inhibitory neurotransmitter that does not normally enter the brain after systemic administration^5^. Here, we show that somatosensory-evoked potentials (SSEP) can be temporarily suppressed by systemically administered GABA in a dose-dependent manner after BBB disruption in the somatosensory cortex.

## Results

**Figure 1:**
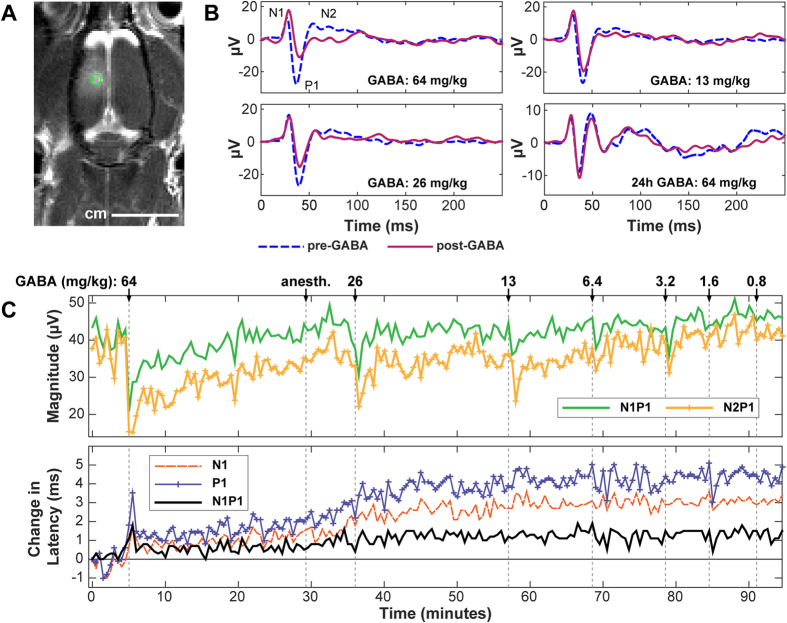
(a) Contrast-enhanced MRI showing ultrasound-induced BBB disruption induced in a rat’s somatosensory cortex in one hemisphere. SSEP recordings were subsequently obtained with needle electrodes placed under the skin at the point indicated by the circle (2 mm lateral, 2 mm posterior to bregma). (**b**) Example SSEP recordings made in Rat 1 before and after administration of different doses of GABA. Measurements were made approximately 40–120 min after FUS-induced BBB disruption and repeated 24 hours later. (**c**) Changes in SSEP magnitude and latency (time-to-peak for N1, P1; time between peaks for N1P1) plotted as a function time in the same rat, who received multiple additional GABA doses over a 90 min period. Changes in latency (in ms) from that measured at the first recording are shown. The SSEP magnitude suppression and the time required to recover to pre-GABA levels were both dose-dependent. Obvious latency changes related to GABA administration were observed only at the highest dose tested. A slow increase in the time-to-peak for both N1 and P1 occurred starting at approximately 30 min. The reason for this increase is not known but may be related to administration of anesthesia a few min earlier.

BBB disruption in the targeted region was evident in MRI as signal enhancement after administration of Gd-DTPA, an MRI contrast agent with a molecular weight of 938 Da that does not normally extravasate into the brain. BBB disruption on the somatosensory cortex was evident in every rat that was sonicated; an example is shown in Fig. 1A. The signal enhancement after Gd-DTPA varied from 6% to 91% (Table 1). In most cases, this disruption covered the entire dorsal-ventral thickness of the brain. BBB disruption was also evident in the thalamus in most animals.

Representative examples of transcranial SSEP recordings obtained during sciatic nerve stimulation before and after intravenous GABA administration are shown in Fig. 1B. Administration of GABA resulted in a dose-dependent suppression of SSEP signals. Figure 1C plots the latency and SSEP magnitude as a function of time during this experiment. Changes in SSEP latency were evident only for the highest dose. A slow increase in the time-to-peak for both N1 and P1 occurred starting at approximately 30 min. The reason for this increase is not known but may be related to administration of anesthesia a few min earlier.

Magnitude suppression was observed after all but the lowest dose. The time required for the SSEP to recover to pre-GABA levels was also dose-dependent. At high concentrations, several minutes were required for the SSEP to recover. At lower doses, the suppression lasted less than a minute. Anomalous changes in SSEP magnitude on the same order as that observed when GABA was administered (such as that in Fig. 1C at approximately 20 min) occurred rarely.

**Figure 2:**
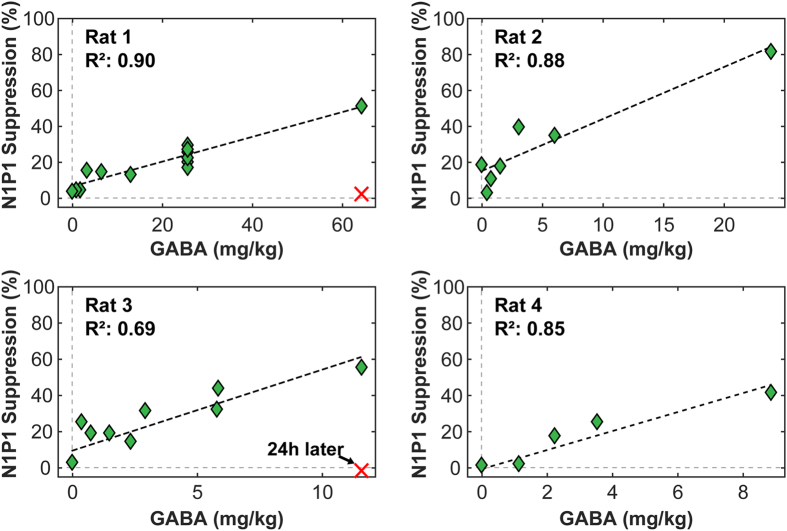
Linear correlation between N1P1 magnitude and GABA dose in Rats 1–4. Ten 26 mg/kg GABA injections were applied over two hours in Rat 1 and produced a repeatable level of suppression. Rats 1 and 3 were tested 24 hours after BBB opening, and no suppression was observed in SSEP recordings.

**Figure 3:**
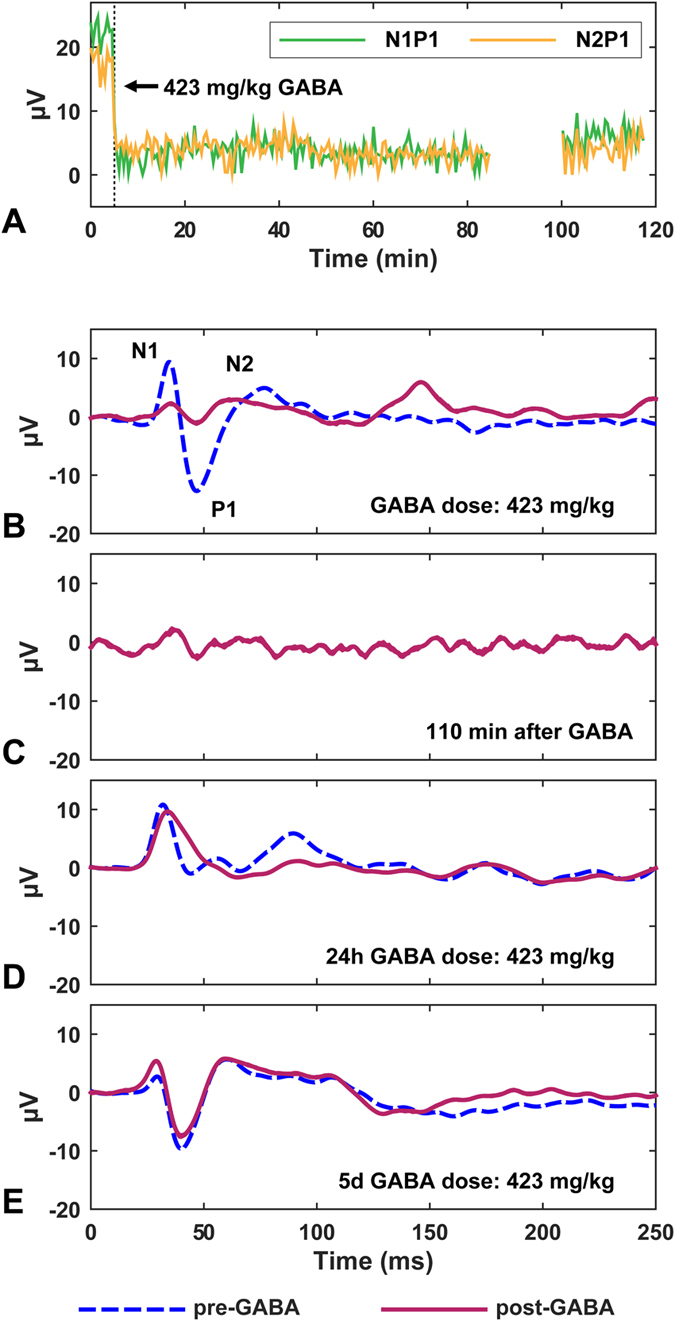
A large GABA dose produced almost complete SSEP suppression for the duration of the experiment – almost two hours. Near the end of the experiment, the measurements were paused for approximately 20 min to confirm that the observed suppression was not due to overstimulation. Minor suppression after GABA injection was observed 24 hours later, but not at 5 days. (**a**) SSEP magnitude vs. time before and after GABA. (**b–d**) SSEP recordings at different times: (**b**) Before and immediately after GABA; **(c**) 110 min later; (**d–e**) Before and after GABA 24 hours and 5 days later.

A linear relationship was observed between SSEP magnitude suppression and GABA dose. Figure 2 shows this relationship for the first four rats who received a range of GABA doses. These slopes, as well as the suppression per mg/kg of GABA for the other four rats who only received one or two injections, are listed in Table 1. When high GABA doses (173–519 mg/kg) were administered, long-lasting suppression was observed. An example is shown in Fig. 3, where the SSEP was almost completely suppressed for approximately two hours. Some suppression was observed 24 h later in this animal, but not at day 5. No suppression was observed in the four control animals who received large GABA doses without BBB disruption. The results from all of the experiments are summarized in Table 1.

## Discussion

GABA is the primary inhibitory neurotransmitter in the brain, and is widely distributed throughout the brain. GABA is released from inhibitory nerve terminals and bound to receptors distributed on post-synaptic cell membranes^6^. GABA’s actions in the synaptic cleft are terminated by its reuptake by either pre-synaptic neurons or nearby glial cells via specific and high-affinity transporters that are believed to be the major mechanism for reducing its concentration in the extracellular fluid^7^. Efflux from the brain to the bloodstream has also been identified as a mechanism that removes extracellular GABA^8^. Such efflux is not significant in the opposite direction, and most studies report that GABA does not cross the BBB^5^. Presumably, FUS-induced BBB disruption enables the passage of GABA from the blood to the brain through opened tight junctions or via intracellular transport^3^ in a quantity that overwhelms this system. It is also possible that efflux mechanisms present in the brain vasculature – the “functional” component of the BBB – are suppressed by the sonication effects.

Previous studies in rats and other animal models indicate that after FUS permeabilization, the BBB remains open with a half-life of 1–5 hours^9^, depending on the size of the injected substance. In this study, consistent suppression was evident for the duration of the experiment (1.5–3.5 hours after sonication), suggesting that the barrier was not restored. In Rat 1, we administered repeated 25.6 mg/kg GABA injections over 165 min and did not observe any attenuation of the suppression. With a large dose, a single GABA bolus injection after BBB disruption resulted in almost complete suppression of the SSEP for at least 110 min (Fig. 3). The plasma half-life of intravenously-injected GABA is approximately 20 min^5^, and the elimination half-life from the brain back into the blood is approximately 17 min^8^. Therefore sustained and potentially more controlled suppression might be best achieved by infusing GABA.

The transducer used in this study had a long focal area that covered the entire thickness of the rat brain in the direction of the ultrasound beam propagation. As a consequence, BBB disruption was produced in both the cortex and subcortical structures such as the thalamus, so we do not know which site(s) were responsible for the suppression we measured. Use of a higher ultrasound frequency could reduce the focal size. We also do not know if the sonications or the BBB disruption itself caused any neuromodulatory effects that influenced the baseline recordings obtained before GABA administration. If any such effects were present, they were not sufficient to prevent us from inducing and recording SSEP. Future work with implanted electrodes, with a FUS system that permits simultaneous electrophysiology, or with functional imaging will be necessary to determine whether such effects were present.

In the present study, the amount of signal enhancement after Gd-DTPA administration varied substantially, even when considering animals imaged at the same magnetic field strength. This variability reflects differences in BBB permeability induced by the sonications and doubtless contributed to the amount of GABA delivered to the brain and the variability in the suppression as a function of GABA dose. It would be interesting to obtain more quantitative MRI measurements^10^ to determine if the amount of Gd-DTPA delivered correlated with the suppressive effects of GABA.

A number of studies have shown that ultrasound alone can suppress brain function^11^,^12^,^13^. Targeted GABA delivery via BBB disruption has several potential advantages over the use of ultrasound alone and other existing neuromodulation techniques. The method is noninvasive, targeted, and can be applied anywhere in the brain, even at deep targets. The use of MRI contrast allows for verification of the location and degree of BBB permeabilization. We can also control the level of the suppression by titrating the GABA dose. Since we are free to sonicate as many targets as we want, we can suppress large regions or multiple targets simultaneously. We can also use different transducer frequencies and geometries to tailor the region to the specific task, including sub-millimeter regions if desired. The mechanisms of the suppression are well understood since the effects of GABA and other neurotransmitters have been studied for decades. Importantly, the method will permit us to transiently block function in a freely behaving subject, even a nonhuman primate^14^, either by recovering the animal from anesthesia after BBB disruption or by performing the sonications in an alert animal^15^. The method is also well-suited for fMRI. Since systems exist that allow for precise, safe transcranial focusing of an ultrasound field in a human^16^ it is possible to use the method in humans. Beyond functional mapping, the method also has potential for surgical planning or even novel therapies.

## Methods

### Animals

All experiments were done in accordance with procedures approved by the Harvard Medical School Institutional Animal Care and Use Committee. The animals were housed, fed, and watered according to the Office of Laboratory Animal Welfare and the Association for Assessment and Accreditation of Laboratory Care regulations. The experiments were performed using male Sprague Dawley rats (262–433 g). Before each procedure, the animals were anesthetized with an i.p. injection of ketamine (90 μg/kg) and xylazine (10 μg/kg) administered hourly or as needed. The fur on the head was removed using clippers and depilatory cream, and a catheter was placed in the tail vein.

### MRI-guided FUS

FUS exposures (10 ms bursts applied at 1 Hz for 60 s) were delivered immediately after the administration of the microbubble ultrasound contrast agent Optison (GE Healthcare) to disrupt the BBB under MRI guidance. The transcranial sonications were applied using 690 kHz FUS transducer driven with a function generator (33220A, Agilent) and amplifier (240L, E&I). Electrical power output was measured using a power meter (E4419B, Agilent) and dual-directional coupler (C5948-10, Werlatone). The transducer was mounted on a manually-operated, three-axis MRI-compatible positioning system. Acoustic coupling between the FUS transducer and the rat’s head was achieved with degassed and deionized water. A transmit/receive surface coil was placed under the rats head, and the system was placed in a clinical 3T (GE Healthcare) or animal 7T (Bruker) MRI. With the 3T MRI, we used a spherically-curved transducer with a diameter and radius of curvature of 10 and 8 cm, respectively. At 7T we used a smaller transducer (diameter/radius of curvature: 4/3 cm). The transducers were calibrated using a radiation force balance to measure the acoustic power and scans of the acoustic intensity were obtained with a 0.2 mm diameter needle hydrophone (HNC-0200, Onda). These calibrations were used to estimate the peak negative pressure amplitude at the focus in water^17^. The width and length of the 50% isopressure contours of the two transducers were 2.3 and 12 mm, respectively, for the 3T transducer; they were 2.3 and 10.3 for the 7T transducer. The transducers, MRI coil, and positioning system were assembled in-house.

Before each experiment, we localized the focal point in the MRI coordinate space by visualizing heating in a silicone phantom (Reston^®^, 3M) using temperature-sensitive MRI. The anesthetized rat was then placed on the system and standard anatomical MRI was obtained to choose the targets. Sonications were applied in a 5 × 2 grid (spacing: 1 mm) centered on the right somatosensory cortex. Each sonication was preceded by a bolus injection of Optison (dose: 200 μl/kg). We waited 1–2 minutes between sonications to allow the bubbles to clear from circulation. After the completion of the ten sonications, axial T1-weighted fast-spin echo images (3T parameters: TR/TE: 500/13.3 ms; echo train length (ETL): 4; field of view (FOV): 8 cm; matrix: 256 × 256; slice thickness: 3 mm; averages: 4. 7T parameters: TR/TE: 600/18 ms; ETL: 4; FOV: 4 cm; matrix: 128 × 128; slice thickness: 1 mm; averages: 4) were acquired before and after an intravenous injection of Gd-DTPA (Magnevist, Berlex), an MRI contrast agent that normally does not cross the BBB. BBB disruption was quantified by signal enhancement after Gd-DTPA injection.

### Electrophysiology

After the sonications, the animals were removed from the MRI system and placed in a stereotactic frame (ASI Instruments). For sciatic nerve stimulation, two wire electrodes (IVES EEG solutions) were inserted dorsally into the left thigh muscle, one near the hip and the other behind the knee. Needle electrodes, constructed from 25-gauge hypodermic needles, were inserted under the skin over the right somatosensory cortex (2 mm lateral, 2 mm posterior to bregma). Reference electrodes were inserted over the olfactory bulb; a ground electrode was inserted into a neck muscle. The sciatic nerve was electrically stimulated with 1 ms bursts at 9–20 V at 1 Hz using a GRASS stimulator (model S48, Astro-Med., Inc.) and stimulus isolation unit (model SIU5 Astro-Med., Inc.) set to capacitive coupling. SSEP recordings were obtained transcranially using a bioamplifier (Octal BioAmp ML138, ADInstruments) and the PowerLab data acquisition system (ML870/P, ADInstruments). Recordings (sample rate: 0 kHz; 30 averages) were obtained using Scope^TM^ (ADInstruments) and averaged to improve the signal-to-noise ratio. The recordings were acquired before and after intravenous GABA at various dosages (Table 1). SSEP recordings were repeated in some animals at 24 h (N = 4) and 5 days (N = 1) after sonication. Control animals (N = 4) received GABA but not BBB disruption.

### Data analysis

Electrophysiology data were processed offline using Matlab (MathWorks). For each SSEP recording, the magnitude and latency (time-to-peak) was determined for the first three peaks (N1, P1, N2). SSEP magnitudes post-GABA injection were calculated as percent changes compared to pre-injection values. The SSEP magnitude change as a function of GABA dose was investigated via least-squares linear regression and calculation of correlation coefficients. The signal change in MRI after Gd-DTPA administration was measured in a 9 × 9 voxel region of interest centered on the somatosensory cortex. For this measurement, the mean enhancement, relative to a pre-contrast image was found. Signal change from a corresponding region of interest in the contralateral hemisphere was subtracted from the measurement to remove contributions from Gd-DTPA in the blood stream.

## Additional Information

**How to cite this article**: McDannold, N. *et al.* Targeted, noninvasive blockade of cortical neuronal activity. *Sci. Rep.* **5**, 16253; doi: 10.1038/srep16253 (2015).

## Figures and Tables

**Table 1:** Experimental parameters and results.

Rat	Pressure amplitude (MPa) in water	GABA		N1P1 Magnitude			N2P1 Magnitude			MRI signal enhancement after Gd-DTPA
		#Injections	Dose (kg/mg)	Max. suppression	% Suppression per mg/kg GABA	Suppression vs. GABA dose R^2^	Max. suppression	% Suppression per mg/kg GABA	Suppression vs. GABA dose R^2^	
1	0.65	12	0.8–64	51%	0.7 ± 0.1	0.90	63%	1.0 ± 0.1	0.81	23%
2	0.64	6	0.4–24	82%	2.9 ± 0.5	0.88	91%	2.7 ± 1.0	0.58	83%†
3	0.65	8	0.3–12	56%	4.5 ± 1.0	0.69	89%	8.6 ± 2.7	0.55	33%†
4	0.60	5	0.5–8.8	42%	5.2 ± 1.1	0.85	34%	6.6 ± 1.1	0.90	28%†
5	0.58	2	173, 519	88%	0.17–0.26	–	83%	0.16–0.39	–	22%
6	0.58	1	423	35%	0.08	–	42%	0.1	–	6%
7	0.58	1	485	38%	0.08	–	63%	0.13	–	30%
8	0.61	1	423	99%	0.23	–	82%	0.19	–	91%
9	Control	1	429	−1%	0.00	–	−9%	−0.02	–	–
10	Control	1	393	7%	0.02	–	9%	0.02	–	–
11	Control	1	179	8%	0.05	–	10%	0.05	–	–
12	Control	1	191	4%	0.02	–	4%	0.02	–	–

^†^7T MRI; other animals were imaged at 3T.
